# Genome-wide association studies in *Plasmodium *species

**DOI:** 10.1186/1741-7007-8-90

**Published:** 2010-07-13

**Authors:** Bridget Penman, Caroline Buckee, Sunetra Gupta, Sean Nee

**Affiliations:** 1 Department of Zoology, University of Oxford, Oxford OX1 3PS, UK; 2 Institute of Evolutionary Biology, University of Edinburgh, Edinburgh EH9 3JT, UK

## Abstract

Genome-wide association studies (GWAS) look for correlations between traits of interest and genetic markers spread throughout the genome. A recent study in *BMC Genetics *has found that populations of the malaria parasite *Plasmodium vivax *should be amenable to GWAS searching for a genetic basis of parasite pathogenicity. Geographical substructure in populations may, however, prove a problem in interpreting the results.

See research article http://www.biomedcentral.com/1471-2156/11/65

## Commentary

Since the publication of the complete human genome sequence in 2003, hundreds of genome-wide association studies (GWAS) have been carried out in the human population to identify polymorphisms associated with human disease [1,2]. As more genome sequences are published, including those of many important human pathogens, there is an expectation that GWAS will also provide insights into the evolution of pathogen virulence or drug resistance.

In a recent study published in *BMC Genetics*, Orjuela-Sanchéz *et al*. [3] present an assessment of the feasibility of future GWAS in the malaria parasite *Plasmodium vivax*. They analyzed a 100-kb chromosome segment (0.4% of the parasite genome) in field samples of the parasite from South America and Asia with the aim of determining the genetic diversity in these populations and to look for geographical effects. The authors found that the Brazilian *vivax *population they studied was amenable to future GWAS as it contained high levels of genetic diversity - a prerequisite for distinguishing the areas of the parasite genome associated with traits of interest. They also confirmed that it exhibits appropriate levels of linkage disequilibrium - the non-random association of specific genetic markers with the trait of interest, indicating that the marker is in or near a gene that underlies that trait. Too little linkage disequilibrium means that a genetic marker is unlikely to stay associated with the gene that causes the trait of interest; too much and there is a risk of 'false positives' where the marker appears correlated with a trait of interest but is actually far away from the gene that governs it. The authors raise several notes of caution, however, not least the fact that substantial geographical substructuring exists in *vivax *populations, which could confound efforts to find true associations between markers and traits. The authors rightly emphasize the need for careful correction for geographical population structure; however, the structuring that they observed among the Brazilian isolates showed no clear relationship with collection site - suggesting that these issues may not be easily resolved by geographical corrections alone.

## What makes a good subject for GWAS?

The first and fundamental consideration in planning a GWAS is how much the trait of interest is likely to be influenced by genetics at all. In the case of a trait such as virulence, it may be that the host's genetics are far more important than the parasite's, and looking at the parasite genome is not going to turn up a useful result. A recent paper by Anderson *et al*. [4] considers how to approach this issue in another malaria parasite, *Plasmodium falciparum*. Here, the authors used microsatellite analysis to determine the relatedness between parasites obtained from 185 patients. Microsatellite analysis is cheap and quick, as appreciated by any daytime TV viewer who has seen it used for talk-show paternity tests. If parasite relatedness correlates with a trait, then parasite genetics are likely to be important in governing that trait. They found that parasite relatedness was correlated with drug resistance, but that the rate of parasite clearance following artemisinin-based combination therapies was not.

Once a trait is identified as having a strong genetic link, we may be justified in conducting a GWAS to look for the genes responsible. However, the very nature of a GWAS presents another problem - this time, statistical. It is well recognized that when processing such large datasets as provided by GWAS, performing many tests looking for associations, there is the high possibility of obtaining an apparently significant association purely by chance. The great evolutionary biologist John Maynard Smith once said: 'statistics is the science that lets you do 20 experiments a year and publish one false result in *Nature*'. There are statistical means for correcting for multiple comparisons - for example, the Bonferroni correction - but these are so stringent that they make it hard to find any effects at all. There is no simple solution to this problem [5]; ultimately we have to rely on the judgement of informed scientists concerning the nature of the genes identified by the analysis. Perhaps this all illustrates that, fortunately, there is no immediate possibility of science being conducted by robots chucking things into sequencers and computer programs analyzing the data and emailing the results to big Pharma.

A third issue to be overcome if a GWAS is to give useful results is the choice of the genetic markers themselves. These markers are single nucleotide polymorphisms (SNPs), and it is vital to choose the correct set of reference SNPs for the question of interest. The older the mutation governing a trait, or the lower the degree of linkage disequilibrium in a population, the higher the density of SNPs required. In malaria parasites, the ratio of self-fertilizing to cross-fertilizing reproductive events is extremely variable in different malaria parasite populations, and is affected by natural selection. This complicates assumptions about linkage disequilibrium across different parts of the genome and increases the density of SNPs required for successful association mapping.

The crucial importance of choosing the correct set of reference SNPs in a GWAS is well illustrated by efforts to explore human genes that influence malaria susceptibility. The mutation responsible for sickle-cell anemia (a point mutation at the beta-globin locus) is known to offer substantial protection against severe malaria (for a review, see [6]). Jallow *et al*. [7] conducted a GWAS of severe malaria in Gambian children, and used the sickle-cell mutation as a benchmark to detect the success of different GWAS methods. When a reference sample from a non-Gambian African population (the Yoruba HapMap sample) was used to analyze the Gambian data, no signal could be detected for the sickle-cell mutation (even though at a macroscopic level the Yoruba and Gambian patterns of linkage disequilibrium appeared similar). Choosing the wrong set of reference SNPs therefore presents a huge potential pitfall for GWAS.

The study by Orjuela-Sanchéz *et al*. [3] hints at the possibility of conserved recombination hotspots within *P. vivax*; such hotspots, as they note, have also been observed in *Plasmodium falciparum *[8]. The identification and confirmation of hotspots will play an important part in the design and analysis of future GWAS in both of these malaria parasites.

## GWAS in *Plasmodium*

The very first *Plasmodium *GWAS was published by Mu *et al*. [9] in December 2009. The authors analyzed 189 *P. falciparum *isolates from Asia (146 isolates), Africa (26 isolates), America (14 isolates) and Papua New Guinea (3 isolates), and conducted a GWAS looking for genes associated with drug resistance. They were able to identify a gene (PFA0665w) that had not previously been associated with the parasite response to antimalarials. As discussed earlier, drug resistance seems to be a trait largely governed by parasite genetics and is thus clearly an area where parasite GWAS may continue to prove helpful. However, as noted by Orjuela-Sanchéz *et al*. [3], gene copy number variation (CNV) can contribute to drug-resistance phenotypes in addition to point mutation. Future GWAS in malaria parasites will need to take CNV into account, and methods are being developed to make this possible [10].

Orjuela-Sanchéz *et al*. [3] make two additional points of broad interest for the GWAS field and beyond. For *P. falciparum*, geographical regions of high transmission have greater genetic diversity than regions with low transmission [11,12]. We expect this relationship because high transmission means higher population sizes of the parasite and lower levels of inbreeding. However, Orjuela-Sanchéz *et al*. identified the opposite relationship between *P. vivax *endemicity and genetic diversity: they observed the highest levels of diversity in their Sri Lankan sample (which came from their area of lowest malaria endemicity), and the lowest in their Vietnamese sample (which came from their area of highest malaria endemicity). Given the aforementioned problems about statistical artifacts in GWAS, such a pattern requires further confirmation - but it is certainly an intriguing result.

Second, their study's investigation of mutations in a known resistance locus found evidence in support of the hypothesis that chloroquine resistance at that locus requires a two-step mutation process. This has previously been suggested by [13] and raises the important possibility using detection of the first mutation as an early warning system for the imminent emergence of drug resistance in a *vivax *population.

It is important to recognize that these are early days in the development of a very exciting new technique, and that it is not surprising that more questions than answers have arisen from its application so far. There are strong analogies here to administering a large questionnaire to a population and hoping that some clear connections will fall out. We know from such experience that patience is necessary, as are continuous refinements of the technique, and continuous revision of the right questions to ask. The pieces of the complex jigsaw may take a long time to clink together, and it is crucial that the faith of the researchers is located in its long-term outcomes.
